# Understanding LSTM Network Behaviour of IMU-Based Locomotion Mode Recognition for Applications in Prostheses and Wearables

**DOI:** 10.3390/s21041264

**Published:** 2021-02-10

**Authors:** Freddie Sherratt, Andrew Plummer, Pejman Iravani

**Affiliations:** Department of Mechanical Engineering, University of Bath, Bath BA2 7AY, UK; F.W.Sherratt@bath.ac.uk (F.S.); A.R.Plummer@bath.ac.uk (A.P.)

**Keywords:** Locomotion Mode Recognition, LMR, HAR, IMU, LSTM, wearables, prosthetic, prostheses

## Abstract

Human Locomotion Mode Recognition (LMR) has the potential to be used as a control mechanism for lower-limb active prostheses. Active prostheses can assist and restore a more natural gait for amputees, but as a medical device it must minimize user risks, such as falls and trips. As such, any control system must have high accuracy and robustness, with a detailed understanding of its internal operation. Long Short-Term Memory (LSTM) machine-learning networks can perform LMR with high accuracy levels. However, the internal behavior during classification is unknown, and they struggle to generalize when presented with novel users. The target problem addressed in this paper is understanding the LSTM classification behavior for LMR. A dataset of six locomotive activities (walking, stopped, stairs and ramps) from 22 non-amputee subjects is collected, capturing both steady-state and transitions between activities in natural environments. Non-amputees are used as a substitute for amputees to provide a larger dataset. The dataset is used to analyze the internal behavior of a reduced complexity LSTM network. This analysis identifies that the model primarily classifies activity type based on data around early stance. Evaluation of generalization for unseen subjects reveals low sensitivity to hyper-parameters and over-fitting to individuals’ gait traits. Investigating the differences between individual subjects showed that gait variations between users primarily occur in early stance, potentially explaining the poor generalization. Adjustment of hyper-parameters alone could not solve this, demonstrating the need for individual personalization of models. The main achievements of the paper are (i) the better understanding of LSTM for LMR, (ii) demonstration of its low sensitivity to learning hyper-parameters when evaluating novel user generalization, and (iii) demonstration of the need for personalization of ML models to achieve acceptable accuracy.

## 1. Introduction

For the non-amputee (in the research field this is commonly referred to as able-bodied, which can be considered an outdated term, so instead, non-amputee will be used in this article), it is taken for granted that during locomotion, both legs will act in unison adapting to the environment and activity without thought; for lower-limb amputees this ability is lost. Amputees suffer from poor gait due to muscle imbalances, and significant compensatory mechanisms are required to adapt to the loss of muscle and joints [1]. This results in musculoskeletal problems, increased energetic cost of locomotion and an increased risk of falling [2,3,4]. The next generation of prostheses aims to replicate the lost power generating functionality of muscles to improve gait. In order for the prosthetic to work in synergy with the user, it must recognize the users intent; therefore, a system of Locomotion Mode Recognition (LMR) is required.

Several commercially available prostheses exist that actively adapt to the user intent, such as Ottobock’s Enpower BiOM [5], Blatchford’s ElanIC [6] and Össur’s Proprio Foot [7]. None of these three provides more than basic functions, such as maintaining dorsiflexion during leg swing to increase toe clearance and adjusting ankle resistance based on terrain. Only the BiOM ankle offers powered assist in push-off, the controller for this relies on hand-tuned heuristics control strategies [8]. The University of Bath with commercial partners has also been developing a next generastion powered prosthesis [9].

Machine Learning (ML) offers the ability to significantly increase the sophistication of such systems, through understanding of a wide range of activities and personalization to individual characteristics, without specialist intervention [10]. As classifying activities is temporal is nature, sequential ML networks, such as Long Short-Term Memory (LSTM), are a good fit. LSTM networks have been demonstrated to be extremely capable at Human Activity Recognition (HAR), accurately identifying actions from locomotive actions, such as Walking, Running and Stairs [11], to Hip-Hop dance moves [12]. However, little is known of their understanding internal behavior during these tasks. For a medical device, such as a prosthetic, both a high levels of accuracy and detailed knowledge of internal network operation is required.

This paper explores in detail the operation and performance of LSTM networks for LMR using both seen and novel users. Data from non-amputee participants is used as a substitute for amputee data as it allows for a much larger and more varied data set while minimizing risk to subjects. This is then used to investigate the internal operation on a simplified LSTM network. The effects of hyper-parameters on the generalization performance of a practical LSTM network are then investigated. Finally, changes to the model are investigated to try and improve its performance for novel users.

The major contributions of this work are as follows:

(1) Methodology for the collection of a large self-supervised data set of human locomotion data in a natural environment.
(2) Provide an insight into the behavior of an LSTM LMR model, and the performance effects of hyper-parameter selection.
(3) Investigation of hyper-parameter sensitivities in an LSTM network and their effect on classification accuracy and generalization to novel user.
(4) Demonstration of the need for personalization techniques to account for individual gait traits.

The remainder of this paper is organized as follows; First background theory on the Human Gait Cycle and LSTMs is presented in Section 2. Section 3 contains Related work followed by Section 4—Materials and Methodology, describing the data collection process and setup of the ML environment. The following Section 5 and Section 6, detail the experiments undertaken, investigating LSTM behavior, and hyper-parameter sensitivities, respectively. These each follow the same structure with an introduction to the experiment, analysis methodology, then results and discussions. The remaining two sections, Section 7 and Section 8, contain discussion and conclusions.

## 2. Human Gait and Machine-Learning Fundamentals

Within this section fundamental theory of the human gait cycle, and Recurrent Neural Networks (RNN) and LSTMs is presented.

### 2.1. Locomotion Mode Recognition and the Human Gait Cycle

**Figure 1:**
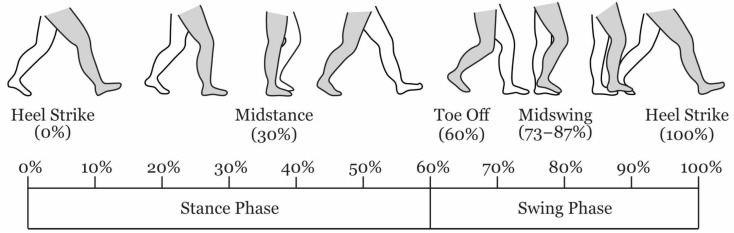
Human Gait Cycle during level walking. The percentage timings of the gait events are approximate, they vary depending on the individual and environment.

Human gait is a cyclic process that can be delineated by key events. A gait cycle is defined by two successive Initial Contact (IC) events (the point at which the foot contacts the ground) of the same limb. As this is normally the heel, it is often referred to as Heel Strike (HS). Conversely, the point when the foot leaves the ground is referred to as Toe Off (TO). These two events are used to subdivide the gait into two phases; stance—when the foot is on the ground, and swing when not. A diagram showing these events and their location in the gait cycles is shown in Figure 1.

**Figure 2:**
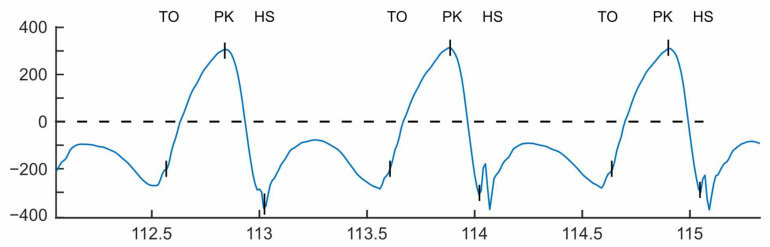
Gait events extracted from the sagittal plane gyroscope signal. IC—Initial Contact, PK—Peak Swing, TO—Toe Off.

It has been shown that gait events can be established from only extrema of the shank angular velocity in the sagittal plane (The sagittal plane divides the body into left and right, so rotation in this plane is forward and backward motion of the shank) using a technique originally presented by Sabatini et al. [13]. IC/HS was found to line up with the minima following the peak swing velocity (PK) and TO was identified as the halfway point between the zero-crossing, negative to positive, and the minima before peak swing. Figure 2 shows the gyroscope trace of a sensor attached to a subject’s shank with the locations of the calculated TO and IC events indicated.

The action of the leg varies depending on the activity. To accommodate this, powered prostheses will require multiple locomotive modes to achieve the different timing and power requirements. Therefore, automated recognition of the user’s intentions and subsequent selection of the corresponding locomotive mode will be crucial to the performance of devices [14,15,16]. In order for amputees to have confidence in a prosthetic device, its activity recognition must be timely, accurate and consistent and able to account for the individual gait characteristics [17,18,19].

For the current generation of prosthetic devices, this is achieved through hand-tuned heuristics. These methods identify and associate changing properties of sensor data with different activities. For example, Coley et al. noted the variation in shank sagittal plane rotational velocity that occur when walking on stairs [20]. It was found that during early stance there is an increase in rotational velocity during stair descent and a decrease during stair ascent when compared to level walking. The current state of the art in LMR uses ML methods to accomplish activity recognition; these techniques will be discussed further in the next section.

### 2.2. Long Short-Term Memory Networks

LMR for active prostheses has conventionally been achieved through heuristic methods with handpicked features that are manually tuned for each individual [21,22]. This approach is favored by the commercial market due to safety and regulatory concerns [23]. The tuning of these controllers is time-consuming and requires a highly skilled prosthetist. In the current state of the art for LMR techniques, the focus has been on the use of ML techniques to automate the process of feature selection, output classification, and personalization [10].

**Figure 3:**
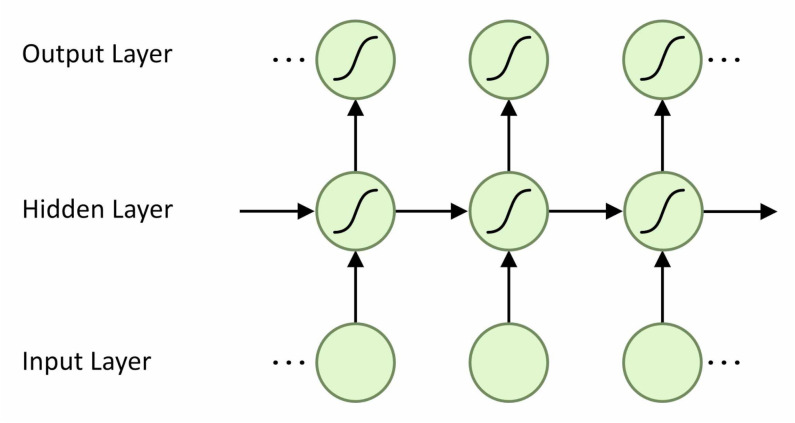
Unfolded Recurrent Network.

Many different machine-learning techniques have been investigated including, Support Vector Machines, Hidden Markov Models and Convolution Neural Networks (CNN) with success [10]. As sensor data from human gait is temporal, the best architecture for solving this will be one that can take into account the sequential nature of the input data. The Recurrent Neural Network (RNN) is an ML architecture suited to handling sequential data as it contains both vertical and horizontal connections. This means that cell activation is related to both the previous time step and the input. Information is therefore passed along the sequence as well as up through layers. Figure 3 shows the unfolded structure of a recurrent network. It can be seen that the activation of each cell is dependent on both its inputs and the hidden states of the previous time steps.

Each timestep in the network can contain several hidden states or units. This is represented by Equation (1) showing the activation input, $a^{t}$. a is formed from the bias vector b plus the sum of input vectors x and previous hidden states h, multiplied by the weight matrices W and U for hidden-to-hidden state and input-to-hidden state connections respectively [24]. The shape of an RNN network is often described by its timesteps and units, for example, 128 × 6.

(1) $$a^{\left(t\right)}=b+{\mathrm{Wh}}^{\left(t-1\right)}+{\mathrm{Ux}}^{\left(t\right)}$$

RNNs have been shown to produce good results in some sequential tasks, but their application is limited by difficulty of training. The primary difficulty is the vanishing/exploding gradient problem. During gradient-based training methods, repeated multiplication by values that are not near one, along long dependency chains results in values that either vanish or explode. A vanishing gradient makes it challenging to know which direction the parameters should move to improve the cost function. Exploding gradients can make learning unstable. Non-gradient-based training has been tried, although to limited success [24,25].

**Figure 4:**
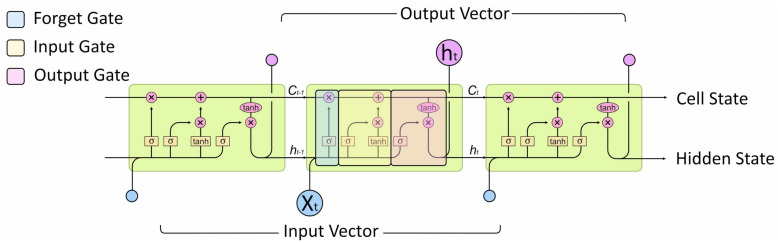
LSTM unit with input and output connections.

The Long Short-Term Memory (LSTM) architecture solves the vanishing gradient problem by adding mechanisms for regulating information allowing it to be retained for long periods. Created by Hochreiter and Schmidhuber in 1997 [26] the LSTM is an RNN style architecture that includes gates to control information flow between cells, see Figure 4. Information flowing along the cell state can be modulated by the input and forget gate structures with the final output a filtered version of the cell state based on context from the inputs [27].

## 3. Related Works

In HAR tasks, LSTMs have been demonstrated to provide exceptional performance [11] although very little work has been done investigating this in the context of prostheses. Labarrière et al. conducted a systematic review of the ML methods used in activity recognition; for assistive device LSTM networks were only used once [10].

LSTM networks have been found to perform highly in HAR and Activities of Daily Living (ADL) tasks. Murad and Pyun investigated Deep LSTM networks for LMR [11]. They trained their network on common ADL datasets, presenting performance in comparison to other ML architectures on the same data sets. The network they used took raw IMU data as its input, then interpreted the data using four LSTM layers before a late fusion dense layer and a SoftMax classifier were used to produce a class output. The number of units in the LSTM layers was not explicitly stated but appeared to be one. Performance is high achieving 96.7% accuracy on the UCI-HAD dataset [28] and an improvement on the presented previous classification attempts using CNN, SVM and other networks. Tufek et al. replicated this result, achieving 93% accuracy on the UCI-HAD data set using only a three-layer LSTM network [29].

However, the accuracy presented is determined from the validation data, a random 20% of the source data, so sufficient separation between training and validation data is not guaranteed. In the compared work, a mixture of evaluation techniques is used, most commonly k-fold cross-validation techniques. With test data selected by leaving out participants [30,31]. As such, it is not clear that a direct comparison can be made to demonstrate LSTM’s superiority.

Different sensor fusion approaches have been tried. Murad et al allowed a deep LSTM network to learn to fuse the sensor modalities [11]. Chung et al. used an ensemble voting arrangement, where each channel modality of sensor data was passed through a separate LSTM network, with a weighted voting system forming the output classification [32]. This achieved a slightly higher accuracy, of 94%, than using the sensors individually.

Multiple authors have developed models that use a series of CNN layer first to fuse sensor data from multiple modalities before passing it to a LSTM network [33,34,35,36,37]. These achieve only minor improvements in performance classification with 95–96% accuracies. Again, none of the authors were clear about the unit shape of their LSTM networks.

There are few examples of LSTM networks being used in assistive devices. Wang et al. used a Deep LSTM network to select locomotion modes for a lower extremity exo-skeleton [38]. Five locomotion modes were classified (sitting, standing, walking and ascending/descending stairs) based on angular information from hip, knee and ankle joints. A two-layer LSTM network with 128 timestep windows was used. The hidden states of this were fed into a weighted mean before a SoftMax classifier. Again, the number of units per timestep was not specified. The classifier performed better than the other models tested achieving over 95%.

Ben-Yue Su et al. presented work investigating intent prediction for trans-tibial amputees using IMU data and a CNN networks [39]. Ten non-amputee and one trans-tibial amputee were asked to perform short walks traversing a short staircase and ramp with a level surface either side. The non-amputee subjects wore a hands-free crutch to simulate amputation. Three IMUs were attached to the thigh, shank and ankle of the “healthy” leg. The CNN classifier identified five steady states and eight transitions between states. An accuracy of 94% was achieved by the non-amputee subjects; this dropped to 89% for the amputee for validation data. When testing generalization to an unseen user, using Leave One Out Cross-Validation (LOOXV), this dropped to 82% for non-amputee subjects. Subject-specific training was recommended. Reasons for poor generalization were not investigated.

Research into the generalization of ML HAR Models to new users is limited. Dehghani et al. investigate the metrics used to evaluate the performance of classifiers, particularly regarding their performance on unseen data presented using k-fold cross-validation methods [40]. The paper implements various forms of ML, such as Support Vector Machines (SVM) and Hidden Markov Models (HMM) but not LSTM. Dehghani found that using validation data to evaluate performance overestimates accuracy by 10–16% as the validation data is too similar to the training data. Instead, individual subjects should be excluded and used as test subjects. The reason for the worse generalization when presented with a novel user has not been investigated.

Investigations into LSTM networks for HAR/LMR have been primarily focused on achieving the highest possible classification accuracy. No one has investigated the internal operation of the network, or sensitivities to hyper-parameter selection for these applications. Dehghani et al. identified that model generalization to novel users is an area that also needs further investigation [40]. This paper aims to address these areas.

## 4. Materials and Methodology

To complete the aims of this paper, a dataset of human locomotion, methods for processing this data and a ML environment are required. This section details the methodology used to provide this. It is split into three sections, Section 4.1 and Section 4.2 detail the data collection and pre-processing, respectively. Section 4.3 presents the ML environment and methods.

### 4.1. Unsupervised Data Collection in Dynamic Natural Environments

There are several commonly used data sets for LMR of non-amputees. The OPPORTUNITY activity recognition dataset [41] contains 18 classes for Activities of Daily Living (ADL) such as opening/closing doors and drinking from a cup. Each subject wore seven 6-axis IMUs and 12 3-axis accelerometers while they performed the prescribed actions. The UCI-HAD dataset [28] recorded subjects performing six activities: walking, stair ascent, stair descent, sitting, standing and lying while wearing a waist-mounted smartphone with onboard Magnetic, Angular Rate and Gravity (MARG) sensors. Both of these data sets were recorded in controlled conditions, so do not capture any variation in the activity that may occur due to the environment. Sztyler and Stuckenschmidt collected data from 15 subjects performing eight activities while wearing six wearable sensors. Recording took place in the same natural environments for each activity. Only steady-state activities were captured and not the transition between them [42]. Due to limitation in the identified data sets, a new set of data is required.

The aim of the new data set was to record natural locomotion in an unstructured environment, capturing both steady-state and the transition between activities across different settings from a wide range of subjects. Collection of large quantities of data from amputees is very challenging, so instead non-amputee subjects are used. Non-amputee subjects have a less varied gait than amputees, but this can be countered by a larger population size.

Non-invasive wearable sensors, such as Inertial Measurement Units (IMU), are an appealing choice for developing such a system. IMUs give fast update rates, 100 s of Hz, are non-invasive (small with minimal mounting constraints), low cost and have reasonable accuracy. They have been widely used in the field, all of the latest generation of powered prosthetic knees investigated by Fluit et al contained IMUs [23].

The Suunto Movesense wearable IMU was used to collect activity data. This is a COTS device containing a nine-axis MARG sensor and a Bluetooth Low Energy (BLE) radio in a small 10 g package. The sensor housing contains a snap connector allowing it to be clipped on attachment hardware. A variety of mounting hardware is available off the shelf. The sensor is user-programmable allowing customized behavior through the Movesense API. To enable the desired streaming application it was programmed to transmit compressed IMU data at 100 Hz over its BLE connection to a custom app running on an android smartphone. The devices come with Factory calibration for the IMU, no additional IMU calibration was undertaken.

**Figure 5:**
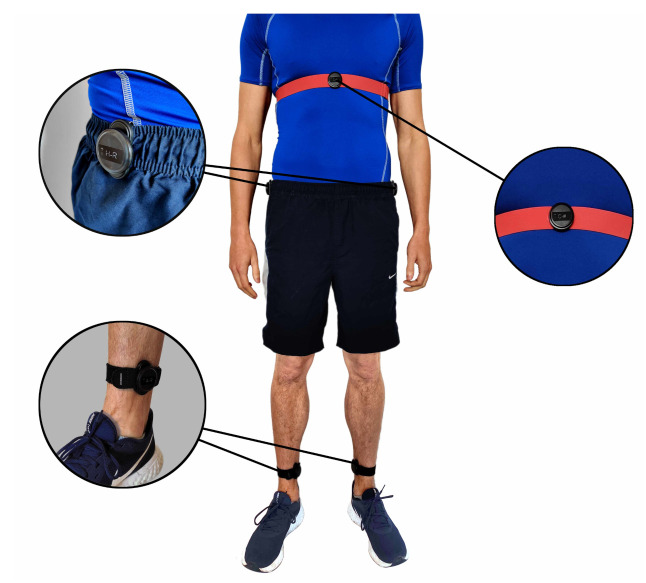
Subject wearing the Movesense IMU sensors on both ankles, hips and the chest.

Five sensors were attached to each participant in the following locations: on the inside of both ankles using an elastic Velcro strap, on each hip using a clothes/belt clip and across the chest using a heart rate strap. The location of the sensors was selected to give wide coverage of body movements while providing easy, secure and non-invasive attachment to minimize discomfort and disruption to natural movement. Figure 5 shows a subject wearing the five sensors.

**Figure 6:**
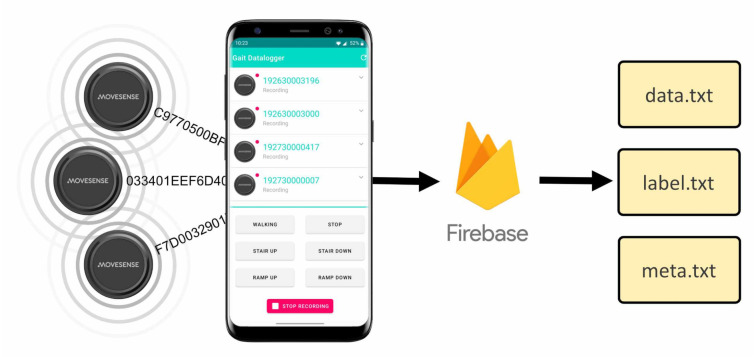
Custom Android app with connected sensors and illustration of Firebase upload system.

To record data from the sensors, a custom android app was created. This formed a BLE connection to each device and saved the streamed data. During recording a series of buttons at the bottom of the screen could be used for real-time labelling of activities. Once recording had finished the subject was presented with an upload screen allowing metadata to be added. The file could then be shared anonymously with the researchers using Google’s Firebase cloud services. A screenshot of the app in recording mode is shown in Figure 6.

Study subjects were provided with instructions on how to use the sensing equipment, and the activity classes, then allowed to record as they wished. The following activities were selected, Walking (W), Stair Ascent (SA), Stair Descent (SD), Ramp Ascent (RA), Ramp Descent (RD) and Stopped (S). Labarrière et al. identified these as the most commonly investigated and they require no equipment or skill to perform [10]. The study received ethical approval from the University of Bath Research Ethics Approval Committee for Health (REACH), reference *EP 19/20 003*.

Twenty-two participants of a wide variety of age (mean 29, std 10), gender (17M, 5F), and physique were chosen to give a broad data set. Participants were instructed to walk around a varied environment with the sensor on while labelling the six activity classes. No further instructions on how the recording should be conducted were provided. A total of 268 min of data was collected, which includes 1170 transitions between activities. Table 1 contains a summary of the data collected. The number of steps was produced by summing the peak swing gait events for each label.

### 4.2. Data Pre-Processing

**Figure 7:**
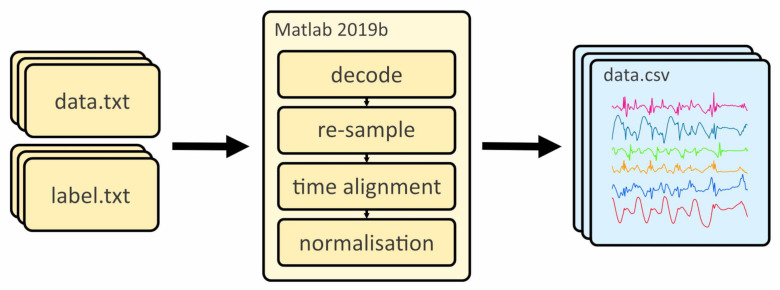
Raw data input and pre-processing flow diagram.

To convert the raw saved data to a form that Tensorflow could import, a processing pipeline was developed in Matlab 2019b. The pipeline consisted of a decoding, re-sampling, time alignment, normalization, and exporting steps. A flow diagram of this process is shown in Figure 7, further details of the process are described below.

The smartphone app does not interpret the compressed data stream, only saving it to a log file. Therefore, the data files need to be converted from a compressed fixed-point form back to their original floating-point representations. This is done by applying the reverse scaling factor to that used by the Movesense device to compress the data. The scaling factor was chosen to provide a balance between accuracy and compression.

To compensate for the difference between the internal sensor clocks the data is re-sampled using the smartphone clock as a common reference. Once a consistent frequency for all the sensor data is achieved, this common reference allows for data from all sensors to be aligned accurately.

Finally, the data is normalized using Equation (2) to scale and shift the data. After this each data channel has a center of zero and standard deviation of one. Normalization is applied on an individual data file basis. In Equation (2) μ is the sample channel mean and μ the sample channel standard deviation. *x* is the input sample and *z* the normalized value. The normalization process removes any overall bias in the IMU data. No additional filtering was applied to the raw data before it was fed to the machine-learning models.

(2) $$z=\frac{x-\mu }{\sigma }$$

The following axis system will be used when presenting and analyzing the results. The axes use a right-hand system with direction, front left and up for *x*, *y* and *z* respectively. *x* is forward towards the front of the body, *y* toward the left and *z* upwards. From this point on, the beginning of the gait cycle, 0%, will be defined as the peak swing maxima. This leads HS by about 20%.

### 4.3. Machine-Learning Methods

Within this subsection, the methodology for setup and training of the machine-learning models are presented. TensorFlow 2.1 was used, with the Keras API used to setup, train and evaluate the different ML Models. The ML environment was developed and run in an Anaconda Python 3 environment. A conventional supervised training setup was used.

#### 4.3.1. Model Setup

Two different model architectures were developed, a simplified model with a single information path for investigating LSTM internal behavior; and a full complexity practical model based on the architecture presented by Murad et al. [11] for investigating hyper-parameter sensitivities. This design was previously discussed in Section 2.2.

**Figure 8:**
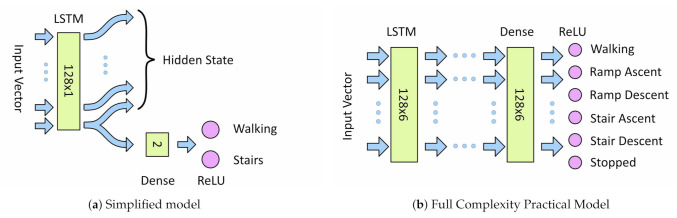
Machine-Learning Model Architectures

For both architectures input data is fed directly into the first LSTM layer. For models with additional LSTM layer, the full output of the first LSTM layer is fed into input of the next layer and so on. The output from the final LSTM layer is fed into a fully connected dense layer followed by a ReLU classifier. For the simplified model the LSTM output is the last timestep only, for the full complexity model the full output of all timesteps is used. The size of the dense layer is equal to the number of outputs from the last LSTM layer. A one-hot classification output is used to encode the activity classes. Figure 8a,b show the architectures of the simplified and full complexity model, respectively.

#### 4.3.2. Data Segmentation

**Figure 9:**
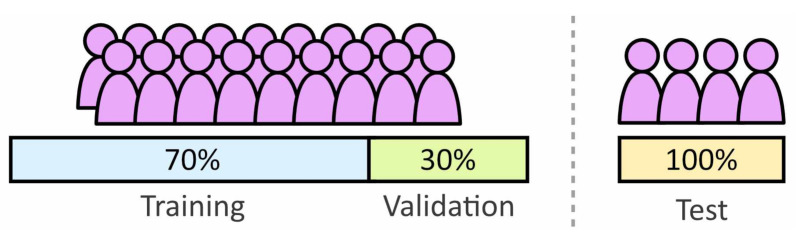
Division of the subject population to form the training, validation and test sets.

The data set was divided into two groups for test and training. The training set was used during the learning process with the test set reserved for evaluating the performance of unseen data. The test set was a variation of Leave One Out Cross-Validation (LOOXV). LOOXV involves training and analyzing the model multiple times with different excluded individuals, the results are then combined to improve statistical certainty. For this paper four/five subjects were excluded each time with analysis repeated five time, meaning each subject was excluded once. The training set contains the remaining subjects, with 30% of the data used as a validation set. Figure 9 provides an illustration of how the data is divided between the three data sets.

To balance the data set, both the training and test sets were adjusted by removing data so that no class contained more than 50% more samples than any another. This re-balancing was undertaken carefully so that during validation splitting the balance was maintained. A class weight input was used to bias the training to further balance the class labels.

The continuous sensor data was segmented using sliding windows. Between the start of each window, an offset of five samples was used. This offset was set empirically to give the model a wide range of data windows position without slowing down learning from an unnecessarily large training set. The output label for each window was set as the recorded ground truth at the end of the window. Classification labels were presented using one-hot encoding.

#### 4.3.3. Model Training

The models were trained to minimize categorical cross-entropy. Model weights were initialized with a Golorot Uniform initializer [43] and optimized with an ADAM optimizer [44]. A dropout of 0.5 was used, selected experimentally, with network connections dropped between the last LSTM output and the dense classifier.

During trained the full training dataset was used for each epoch, with data passed to the optimizer in mini batches of 2000 windows. At the end of each epoch the entire validation set was evaluated. Early stopping was used to prevent over-fitting, this stopped training when stagnation of validation cross-entropy loss was observed. Stagnation was identified by three consecutive losses of greater than the minimum previously seen.

The model was trained on a PC with an AMD Ryzen 3600 CPU and a Nvidia Geforce RTX 2060 Super. Using GPU training, each epoch took approximately 10 s with between 30 and 100 epochs required to train each model depending on the model size and the number of output classes.

## 5. Investigation of LSTM Behavior

An understanding of the internal operation of an LSTM LMR network is important in assessing the network limitations. To capture the internal behavior the effects of input data on the output must be established. This will be achieved by mapping changes in internal hidden state to incoming data.

This analysis can only be performed on low-complexity networks, as information paths of large networks become too convoluted making tracing infeasible. The experiments will use the simplified model, described in Section 4.3.1, as this only has one path for information to flow along. For the simplified network analysis only a single shank IMU sensor will be used. From visual inspection this showed the most variation between activities and subjects.

### 5.1. Analysis Methodology

To enable changes in the hidden state to be mapped to features of the input data, typical plots of input sensor data for different activities are required. This will also allow differences between individual’s gait to be assessed. A typical gait cycle was produced by combining multiple gait cycles for different activities. Each gait cycle can then be normalized to percentage gait, with the mean and standard deviation of multiple cycles plotted to produce activity trends.

Using an extraction of the hidden state, a measure of information gained from the input data will be drawn. Due to reduced learning capacity of the model, in order to get a meaningful classification accuracy, the classification is performed on a reduced number of classes. The data labels are reduced to include only the three most prevalent activities (Walking, Stair Ascent and Stair Descent). The total output classes can be reduced further by combining pairs of these.

Four different combinations of output class for the three activities were tested with four different combinations of input sensor, *y* Gyroscope, *x* Accelerometer, *y* Gyroscope and *x* Accelerometer, and a full six-axis IMU. The *y* gyroscope and *x* accelerometer were selected as visually they showed the greatest variation between activities.

To extract the hidden state the weights and biases of the trained LSTM layer were extracted and copied into a new model whose output was the full hidden state sequence. Input data was then fed into the new network to extract the hidden state. To observe patterns in the hidden state, multiple data windows were overlaid. Variations in step cadence were removed by normalizing to gait cycle. Different activities were then plotted independently to show how the network acts to each. The hidden state output is shown on the *y* axis. This is a dimensionless value which tends toward a value depending on the network classification decision. The value is dependent on the classifier weights but is typically −1, 0 or 1. The final element of the hidden state is fed into the dense layer which forms a classification based on its learnt thresholds.

### 5.2. Results and Analysis

Below the results of the experiments investigating the internal behavior of a LMR LSTM network are presented and analyzed.

#### 5.2.1. Individual’s Gait Trends

**Figure 10:**
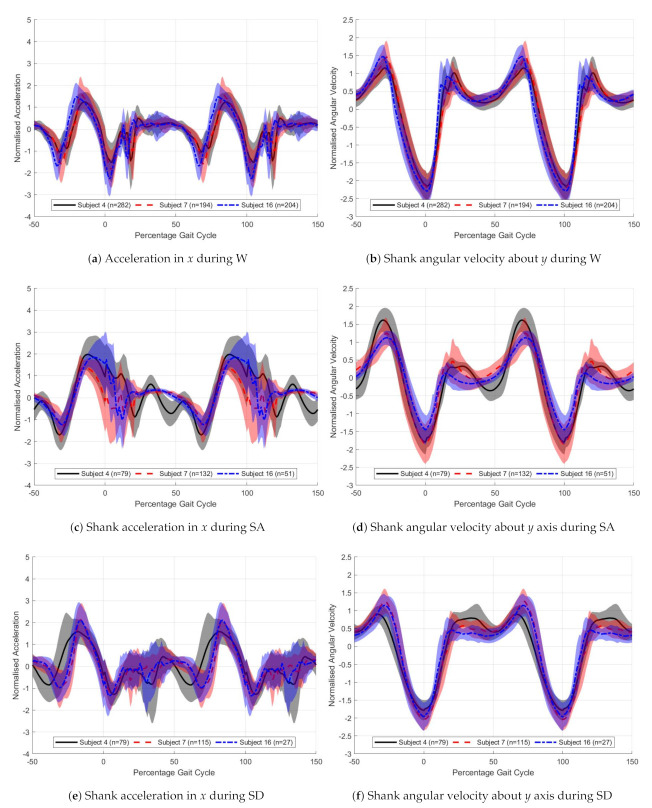
Gait trends for the right shank x accelerometer and *y* gyroscope for 3 different activities. The solid lines show the mean and the shaded area the standard deviation for n steps. Black solid—Subject 4, Red dashed—Subject 7, Blue dot-dash—Subject 16.

Figure 10 show the typical sensor data trends for different individuals and activities. The solid lines represent the mean and the filled area the standard deviation. the *x* accelerometer and *y* gyroscope signals, for three different activities W, SA, SD. On each plot three individuals have been super-imposed over each other. 0% gait cycle corresponds with the peak *y*-axis shank angular velocity.

From Figure 10 the differences between the three chosen participants can be seen. The *x* acceleration signal is very noisy, with large standard deviation seen particularly around heel strike, 20% gait cycle. Smoothing of the input data could be used to reduce this, but this was not investigated. The gyroscope signals are more consistent, shown by the reduced standard deviation.

The stance angular velocities match the results presented by Coley et al. [20]. Stair ascent has a lower early stance rate and stair descent a higher. For stair ascent, there is a delayed peak acceleration with stair descent and walking having very similar shapes. The difference in peak magnitude between activities is a result of variation in step cadence.

The variations in sensor value between subjects is less than the variations between activities, with early stance having the greatest variations between participants for both sensors plotted. Walking shows the most consistent results among participants. These trends hold true for the subjects not shown.

#### 5.2.2. Simplified LSTM Model Behavior

Table 2 presents the classification accuracies of each input and class combination. For each model, classification accuracy was recorded for the validation data and a set of unseen test data from excluded participants. It can be seen that all the models performed equally well for both validation and test data sets. Given the simplicity of the models, this suggests that an LSTM can separate activities from only the prominent features for both seen and unseen users, but only to around 80% accuracy.

The models struggled to separate stair descent from the other two activities and, apart from with the six-axis IMU, most accurately classified stair ascent. All models performed worst when attempting the hardest task of classifying all three activities individually. An input of the *x* accelerometer on its own performed most accurately, even compared to models with multiple input sensor channels. When using only the *y* gyroscope, it was not possible to separate the three activities individually.

**Figure 11:**
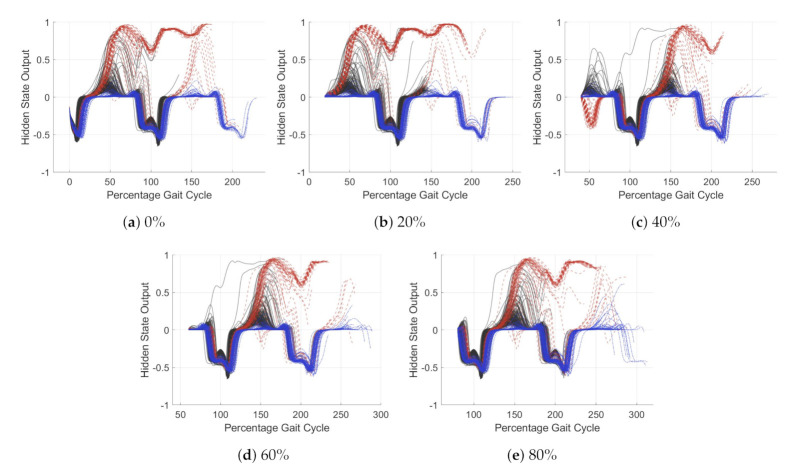
Hidden state of single unit LSTM model with *x* axis accelerometer as its input. The model output classifies stair ascent from walking and stair descent. walking (solid black), stair ascent (dashed red) and stair descent (dot-dash blue). The *x* axis represent the percentage gait cycle, the *y* axis is the dimensionless hidden state value, this tends to 1 for stair ascent and 0 for walking and stair descent.

**Figure 12:**
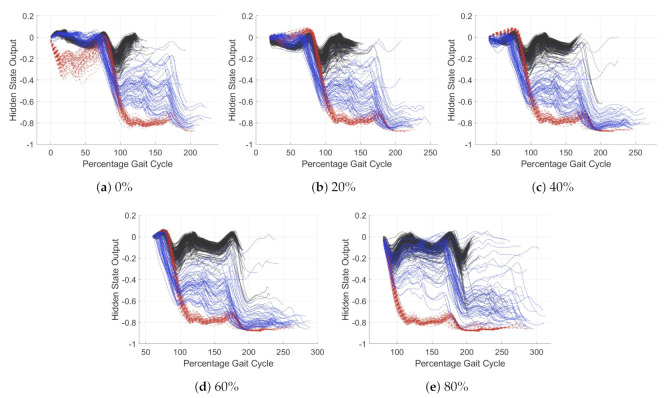
Hidden state of single unit LSTM model with *x* axis accelerometer as its input. The model output classifies walking from stairs (ascent and descent). walking (solid black), stair ascent (dashed red) and stair descent (dot-dash blue). The *x* axis represent the percentage gait cycle, the *y* axis is the dimensionless hidden state value, this tends to 0 for walking and −1 for stair ascent and descent.

Figure 11 and Figure 12 show the trends in hidden state for the simplified model at different percentage points through the gait cycle. Figure 11 has an input of the *y* axis gyroscope and Figure 12 the *x* axis accelerometer. In Figure 11, the model is classifying stair ascent from a combined class of stair descent and walking. For Figure 12, the model is classifying walking from stairs (ascent and descent). Each of the activities is plotted in a different color, solid black for walking, dashed red for stair ascent and dot-dash blue for stair descent. The five subplots show the windows starting at different percentage offset from peak swing. The *x*-axis has units of percentage gait cycle, the *y* axis is the dimensionless output of the hidden state. Values of the *y* axis tend towards −1, 0 or 1, depending on the dense layer classifier weights. A value close to these represents a more certain classification.

For both acceleration and gyroscope, the hidden state value changes most during early stance. For the *y* gyroscope, Figure 11, it can be seen that the classification of stair ascent from walking and stair descent occurs around 50% gait cycle. For the *x* accelerometer, Figure 12, this occurs later in the gait cycle, around 70%. For the *x* accelerometer hidden state trends are less tightly grouped, likely due to noisy input data. This may be fixed by input smoothing, further work is required to investigate this. Classification of stair descent is less certain; the model struggles to separate this from the other two classes. Stair ascent and walking are easily classified.

The simplified model is very good at adapting to variation in gait cadence. This can be seen as despite the steps plotted being normalized to gait cycle, the trends in LSTM hidden state were consistent. This suggests there is little need to adjust the input data to account for variations in cadence.

Analysis of the simplified model has demonstrated that even a model of extremely limited learning capacity can achieve reasonable LMR classification accuracy. It has also shown that the classification of activity occurs exclusively within the early stance phase for the three activities examined. This suggests that the model will be highly sensitive in this area. The model also obtained minimal additional information beyond one stance period; therefore, a window of greater than one gait cycle is unnecessary. The learning from this will now be compared to a full complexity model to verify the results broader applicability.

## 6. Practical LMR LSTM Network Hyper-Parameter Sensitivities

Within this section, the effect of hyper-parameter selection on model performance for a practical LMR LSTM network will be evaluated. The network architecture used is described in Section 4.3.1.

The following hyper-parameters will be investigated: Window size, LSTM units, Number of layers, Different Sensor inputs, and Number of training subjects.

Finally, a simple attempt to improve performance around the transition region will be assessed. The transition between activities is highly variable, data label augmentation will be investigated to add a seventh output, transition, to try and identify this area and act as a measure of confidence.

### 6.1. Analysis Methodology

For the more complex models, the fully connected links between layers and the hidden state become too convoluted to interpret directly. Instead, classification accuracy will be the primary measure of model performance. This will be given as the percentage of correctly classified windows out of the total input windows. Validation data will be used to evaluate seen data performance, and used test data as a measure of generalization to unseen data.

To investigate the network dimensions, three different window lengths (32, 64 and 128 timesteps at 100 Hz), and six unit widths (4, 6, 8, 16, 32, 64) will be tested. For each model shape, the model was trained five times for the five different train/test data sets. With performance evaluated by classification accuracy.

To evaluate how the number of training subjects effects the performance of the model, models were trained with varying numbers of individuals. Between one and 21 training subjects were tested, with a single subject used as the test set. For each incremental increase in subjects, the model was trained ten times with a different excluded subject.

Miss-classification will be analyzed using confusion matrices. A confusion matrix is a tabular representation of the performance of a classifier. Each cell is populated by a count of the ground truth against the classified output. This allows the accuracy of individual classes and confusion between classes to be assessed.

The time series classification output is also used to identify regions of particular uncertainty. By plotting ground truth and classifying labels as color-coded regions on a time axis, areas of incorrect classification can be assessed.

Finally, the seventh classification output, transition, will be evaluated. To train the model for this, data labels will be augmented with a transition region added for 0.5 s before and after changes in activity. Models of varying hyper-parameters will then be trained with the transition state in and classification accuracy used to evaluate performance.

### 6.2. Results and Analysis

Below the results of the experiments on the practical LMR LSTM network are presented and analyzed.

#### 6.2.1. Network Size

**Figure 13:**
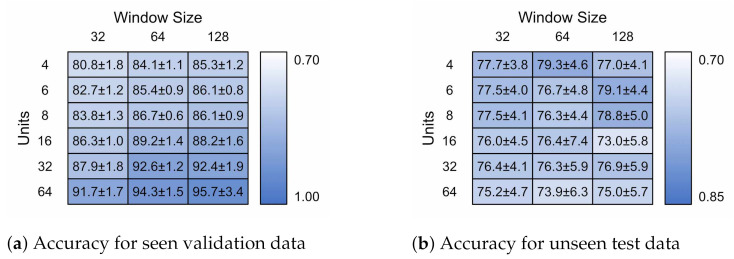
Model accuracy for hyper-parameters of different LSTM units and input window size for both seen and novel subjects.

Figure 13 presents the model accuracies achieved for each model ± standard deviation (*n* = 5). Figure 13a,b contain the validation and test classification accuracies, respectively. The 32 × 4 model contained 1124 parameters, the 128 × 6 model 67,334 parameters.

The validation accuracy increases with increasing model size. The improvement when moving from a 32 timestep window to 64 is much greater than when increasing to 128. The number of units was the most direct factor in achieving higher validation accuracy. For test accuracy, the results plateau around 80%, after which the improvements in validation performance likely occur due to over-fitting to individual traits of the training participants. This also corresponds with an increase in standard deviation for the test data set. A complete gait cycle takes approximately one second, 100 timesteps, so it is likely that exceeding this would make little difference. The continuing performance is likely due to the larger, more sophisticated dense layer.

Multi-layer networks were also investigated. Networks with two, three and four deep LSTM layers were tested, but they showed no improvement in generalization and only a small improvement in validation accuracy. The same was observed with multiple sensors; there was no additional improvement in generalization beyond a single 6-axis shank IMU, only an improvement in seen data accuracy. When the three sensor locations were tested individually the shank IMU performed best.

#### 6.2.2. Number of Training Subject

**Figure 14:**
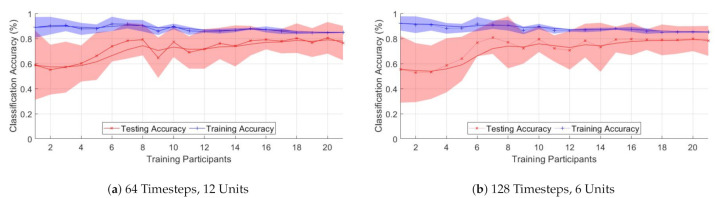
Classification accuracy of seen/unseen subjects for training with different numbers of participants for two different models.

Figure 14 presents the changes in accuracy for varying numbers of training participants. The red line represents the smoothed unseen test subject classification accuracy average for the ten models trained. The blue line represents the same for the validation data. The solid area represents the standard deviation at each point. Figure 14a,b show the results for a 64 timestep 12 unit and 128 time step 6 unit models, respectively.

Figure 14 shows that increasing the number of participants leads to better generalized performance; however, the effects on increasing numbers of participants levels off at around 15 participants. This would indicate that for novel subjects to achieve high levels of classification performance, increasing the number of subjects alone may not be enough.

#### 6.2.3. Analysis of Miss-classification

**Figure 15:**
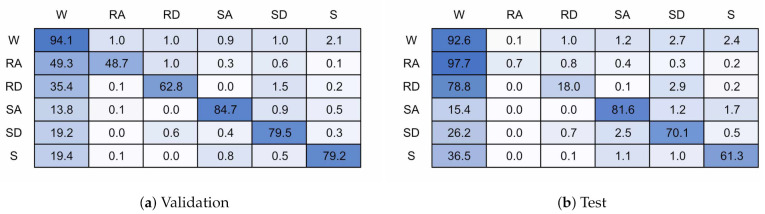
128 timestep, 6 unit confusion matrices.

**Figure 16:**
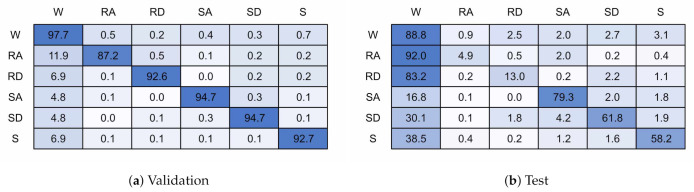
128 timestep, 32 unit confusion matrices.

Figure 15 shows the confusion matrices for a 128 timestep, 6 unit single layer LSTM network. Figure 15a is for the training validation data and Figure 15b the unseen test data. Figure 16 shows the same for a model with 128 timesteps and 32 units. The 6 unit model had an overall classification accuracy of 87.4% for validation and 84.7% for test, the 32 unit model accuracy was 96.1% and 76.0%.

It can be seen that nearly all miss-classifications are confusions with walking. The test data showed a similar pattern for both models, even though the 32 unit model is over-fitted to the training participants. Ramp descent and ascent are both heavily confused with walking. This is likely because of the similarities in gait cycle between the two activities, and the difficulty is accurately labelling this activity due to subject biases. Stairs get slightly confused between each other but again mostly with walking. Stair Descent performs worse than stair ascent. It is not obvious why stop performs poorly, although possibly due to limited data. Some miss-classifications may have occurred due to under labelling or inaccuracies in labelling during recording.

**Figure 17:**
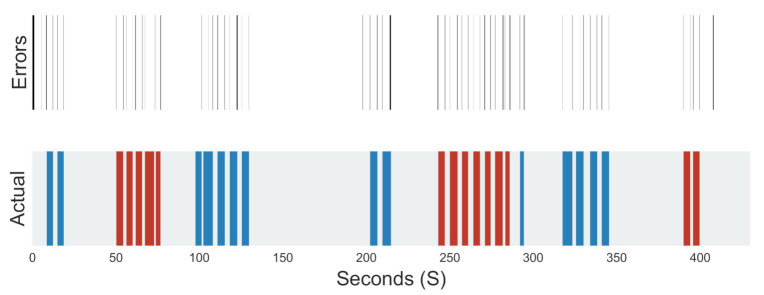
Miss-classifications and labelled activity locations, grey—walking, red—stair ascent, blue—stair descent.

Figure 17 shows a visual representation of the activities labelled during a recording, above which is a plot of where the classification errors occurred. As can be seen, a large proportion of miss-classification occur around changes in activity. This is likely because the transition between activities is highly variable and uncertain.

#### 6.2.4. Transition State

**Figure 18:**
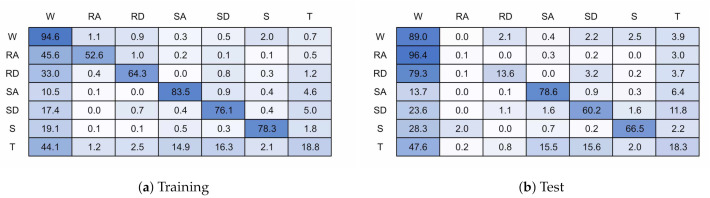
128 × 6 Transition Model.

**Figure 19:**
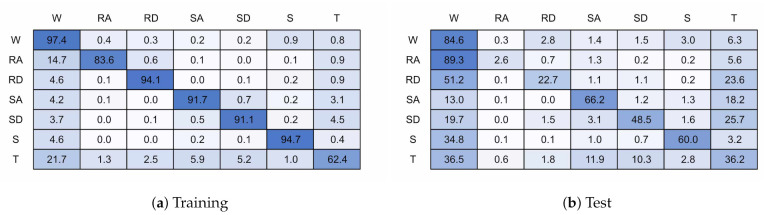
128 × 32 Transition Model.

Figure 18 and Figure 19 present the confusion matrices for the transition models trained with 6 and 32 units, respectively. The 6 unit model achieved 82.8% accuracy on validation data and 72.4% for test data, and the 32 unit model achieved 93.1% and 69.3%. If the transition state is excluded from the classification accuracy then when presented with test data the models achieve 75.2% and 75.0% accuracy for the 6 and 32 unit models, respectively.

The addition of a transition state has not improved classification performance, achieving equal or worse performance than without the transition state, even when excluding the transition state for classification accuracy. This result is unexpected and requires further investigation but at first impression this is due to the transition region being highly uncertain, and so it appears the model cannot map this into a single state. Further work is required to investigate if there are other methods of determining model uncertainty.

## 7. Discussion

The study set out to understand the operation of an LSTM LMR network and the effects of its hyper-parameters on classification accuracy and generalization.

Analysis of the simplified model identified that early stance was a prominent feature in the separation of walking from stair ascent and descent, suggesting a high model sensitivity in the early stance region. It was also observed that this was the area of most variation between individuals. Hyper-parameter sets that achieved greater than 80% accuracy reduced the performance of the classifier on unseen data. This suggests that it was over-fitting to individual subject’s gait traits reducing generalization. The larger standard deviation in the test set also points to this conclusion. Adjustment of hyper-parameters and standard regularization techniques alone were not sufficient to solve this over-fitting. These two observations may begin to explain the challenges in achieving greater than 80% classification accuracy when presenting novel users to the model. This expands on the observation by Dehghani et al. [40] by suggesting that instead of a 15% reduction in performance with unseen subjects, there is a maximum ceiling of performance of around 80%.

When investigating hyper-parameters, the model was able to demonstrate high levels of performance on the created dataset. Classification performance was comparable to literature achieved similar accuracies to the best performing model. It is noted that due to few studies being forthcoming on the exact network units, found to be a very important hyper-parameter, direct comparison with literature is challenging.

Prediction of class around transition was challenging, and the addition of a class for this region did not help. Investigation of other methods to solve this is required, such as a form of output averaging or methods for gauging uncertainty from the full classifier output.

Increasing the number of participants in the study did not improve generalization beyond around 80%. It can be theorized that this would only help if the model were trained on a subject with a similar gait to that of the novel user. As amputees have much more varied gait, this approach is unlikely to be practical. There is the potential that a form of data-augmentation may help with this, but this has not been investigated. A more realistic approach is likely a form of individual personalization.

Implementing these techniques in a prosthetic device is still a way off. The results show promise, but further studies are required to address the concerns raised. Practical considerations are also needed; the LSTM model created has a small parameter count, but still requires many calculations to be implemented on an embedded system. Suitable fail safes would also be required to ensure no harm came to the user, especially if it had not been trained to their gait.

The study was limited to non-amputee data to achieve a large enough population. Data from 22 subjects was collected; this is a large data set, but still a relatively small study. It is also still to be determined how applicable the outcomes of this study are to amputees.

The investigation into hyper-parameters was broad, but there are still many more that could have been investigated. Such as the use of IMU sensors from different locations and the filtering of the IMU data.

## 8. Conclusions

Within this paper we explored the behavior of an LSTM network trained to complete LMR tasks. In literature, there is a lack of studies investigating the internal operation of an LMR LSTM networks, their hyper-parameter sensitivities and poor novel user generalization.

A new dataset for LMR research of 22 non-amputee subjects performing six activities in a real-world environment was collected. A comparison of sensor data for the gait of different subjects revealed that most variability occurred in early stance. Using the dataset, the behavior of the LSTM layer was examined though mapping input data to changes in hidden state. This revealed that the model primarily classified based on data around early stance. This behavior could only be directly observed on a simplified model, as the full-connected nature of a practical network makes it too convoluted to interpret hidden state.

A practical LMR LSTM network was trained for a wide variety of hyper-parameter values to determine its sensitivities. Classification accuracy, of both validation and novel users test data, was used to determine the generalization performance. This revealed that although the network can potentially achieve >95% accuracy, it is over-fitting to individuals gait traits. This is likely due to the model sensitivities in the most individually variable phase of the gait cycle. There is also an increase in erroneous classification around the transition between activities. None of the hyper-parameters tested were able to account for these issues.

The paper shows that network size, number of individuals training data, and number and location of sensors make insignificant contribution to network generalization performance, demonstrating that personalization is critical.

The outcomes of this work suggested that in order to achieve acceptable accuracy rates (above 95%) for novel users, a form of model personalization is required. Additionally, measures to mitigate the errors around transitions are required. Finally, testing with amputee data is required to determine the applicability of the results to prostheses.

## Figures and Tables

**Table 1:** Quantity of data collected for each activity.

Activity	Samples	Time (min)	Number of Steps
Walking	1,075,211	179	9438
Stair Ascent	139,922	23	1286
Stair Descent	122,379	20	1280
Ramp Ascent	73,328	12	656
Ramp Descent	79,436	13	754
Stop	121,027	20	-
**Total**	1,611,303	268	13,414

**Table 2:** Summary of simplified model performance.

Model Classes	Sensor	Validation Accuracy	Test Accuracy
W, SA+SD	*y* Gyroscope	71.6%	86.0%
SA, W+SD	*y* Gyroscope	82.7%	83.7%
SD, W+SA	*y* Gyroscope	57.9%	65.6%
W, SA, SD	*y* Gyroscope	*	*
W, SA+SD	*x* Accelerometer	86.6%	89.2%
SA, W+SD	*x* Accelerometer	88.6%	87.1%
SD, W+SA	*x* Accelerometer	78.4%	81.9%
W, SA, SD	*x* Accelerometer	71.9%	72.4%
W, SA+SD	*x* Accel and *y* Gyro	59.3%	48.8%
SA, W+SD	*x* Accel and *y* Gyro	71.2%	67.1%
SD, W+SA	*x* Accel and *y* Gyro	75.1%	80.8%
W, SA, SD	*x* Accel and *y* Gyro	58.6%	66.2%
W, SA+SD	6 axis IMU	82.0%	83.3%
SA, W+SD	6 axis IMU	74.2%	71.8%
SD, W+SA	6 axis IMU	55.3%	63.3%
W, SA, SD	6 axis IMU	48.0%	50.7%

* Unable to train a model that could classify this set of classes.

## Data Availability

The data generated during this study is available to download from Zenodo, https://doi.org/10.5281/zenodo.4390498 (accessed on 22 December 2020), under the Creative Commons Attribution 4.0 license.

## Abbreviations

The following abbreviations are used in this manuscript:

ADL	Activities of Daily Living
BLE	Bluetooth Low Energy
COTS	Commercial Off The Shelf
HS	Heel Strike
HAR	Human Activity Recognition
HMM	Hidden Markov Model
IC	Initial Contact
IMU	Inertial Measurement Unit
LMR	Locomotion Mode Recognition
MARG	Magnetic Angular and Rate Gyroscope
ML	Machine Learning
LOOXV	Leave One Out Cross-Validation
LSTM	Long Short-Term Memory
RA	Ramp Ascent
RD	Ramp Descent
RNN	Recurrent Neural Network
PK	Peak Swing
S	Stop
SA	Stair Ascent
SD	Stair Descent
SVM	Support Vector Machine
TO	Toe Off
W	Walking
